# Impact of early intravenous amiodarone administration on neurological outcome in refractory ventricular fibrillation: retrospective analysis of prospectively collected prehospital data

**DOI:** 10.1186/s13049-019-0688-1

**Published:** 2019-12-10

**Authors:** Dong Keon Lee, Yu Jin Kim, Giwoon Kim, Choung Ah. Lee, Hyung Jun Moon, Jaehoon Oh, Hae Chul Yang, Han Joo Choi, Young Taeck Oh, Seung Min Park

**Affiliations:** 1 Department of Emergency Medicine, Seoul National University Bundang Hospital, 13620 82, Gumi-ro 173 Beon-gil, Bundang-gu, Seongnam-si, Gyeonggi-do Republic of Korea; 2 Department of Emergency Medicine, Soonchunhyang University Bucheon Hospital, 170 ,Jomaru-ro, Wonmi-gu, Bucheon-si, 14584 Gyeonggi-do Republic of Korea; 3 Department of Emergency Medicine, Hallym University Dongtan Sacred Heart Hospital, 7, Keunjaebong-gil, Hwaseong-si, 18450 Gyeonggi-do Republic of Korea; 4 Department of Emergency Medicine, Soonchunhyang University Cheonan Hospital, 31, Suncheonhyang 6-gil, Dongnam-gu, Cheonan-si, 31151 Chungcheongnam-do Republic of Korea; 5 Department of Emergency Medicine, College of Medicine, Hanyang University, 222-1, Wangsimni-ro, Seongdong-gu, Seoul, 04763 Republic of Korea; 6 Researcher, Seoul National University Bundang Hospital, 82, Gumi-ro 173beon-gil, Bundang-gu, Seongnam-si, 13620 Gyeonggi-do Republic of Korea; 7 Department of emergency medicine, Dankook University College of Medicine, 201 Manghyang-ro, Dongnam-gu, Cheonan-si, 31116 Chungcheongnam-do Republic of Korea

**Keywords:** Cardiopulmonary resuscitation, Emergency medical services, Ventricular fibrillation, Amiodarone, Prognosis

## Abstract

**Background:**

The 2015 AHA guidelines recommend that amiodarone should be used for patients with refractory ventricular fibrillation (RVF). However, the optimal time interval between the incoming call and amiodarone administration (call-to-amiodarone administration interval) in RVF patients has not been investigated. We hypothesized that the time elapsed until amiodarone administration could affect the neurological outcome at hospital discharge in patients with RVF.

**Methods and results:**

This study is a retrospective analysis of prospectively collected data. One hundred thirty-four patients were enrolled. In univariate logistic regression, the probability of a good neurological outcome at hospital discharge decreased as the time elapsed until amiodarone administration increased (OR 0.89 [95% CI = 0.80–0.99]). In multivariate logistic regression, the patients who were administered amiodarone in less than 20 min showed higher rates of prehospital ROSC, survival at hospital arrival, any ROSC, survival at admission, survival to discharge, and good CPC at hospital discharge. The call-to-amiodarone administration interval of ≤20 min (OR 6.92, 95% CI 1.72–27.80) was the independent factor affecting the neurological outcome at hospital discharge.

**Conclusion:**

Early amiodarone administration (≤ 20 min) showed better neurological outcome at hospital discharge for OHCA patients who showed initial ventricular fibrillation and subsequent RVF.

## Introduction

The out-of-hospital cardiac arrest (OHCA) patients who present with initial shockable rhythm (ventricular fibrillation [VF], pulseless ventricular tachycardia) have a good prognosis [1, 2], but early defibrillation is crucial for these patients [3]. However, there are patients who do not respond to electrical defibrillation and present ‘refractoriness’ (persistent VF after more than three defibrillation attempts) [4, 5]. Sakai et al. reported that the age-adjusted incidence rate of shock-resistant VF was 0.4–0.6 per 100,000 people per year [6].

The 2015 American Heart Association guidelines recommend amiodarone to be used on patients with refractory VF (RVF) [7]. Amiodarone is a class III antiarrhythmic agent (potassium channel blocker) in the Vaughan-Williams classification. It affects ion channels, receptors, and sympathetic activity. It terminates VF by blocking the potassium currents, sodium and calcium channels, and α- and β-receptor actions [8]. However, Kudenchuk reported that amiodarone administration resulted in a higher survival to admission rate without the improvement of survival at discharge or neurologically intact survival in RVF patients [9].

Regarding epinephrine, a correlation between the time of epinephrine administration and the neurological outcome was observed, with early intravenous (IV) epinephrine administration resulting in a better neurological outcome in OHCA with VF [10]. However, there has been no study on the time of amiodarone administration and its association with neurological prognosis.

Therefore, we hypothesized that the time elapsed until amiodarone administration could affect the neurological outcome at hospital discharge in patients with RVF.

## Method

### Study protocol

We performed a retrospective analysis of prospectively collected OHCA data. In South Korea, when a suspected cardiac arrest victim was reported to emergency medical services (EMS), at least two emergency medical technician (EMT) teams are dispatched to perform ALS under the emergency physician’s medical control with a video call. The two CPR teams are consistent with a total of 4 to 6 persons, including at least two level-1 EMT and the others were Level-2 EMT or nurses. The level-1 EMTs correspond to EMT paramedics in the United States of America; they are allowed to insert an IV catheter and an advanced airway tube but only under an emergency physician’s medical control. Level-2 EMTs correspond to EMT basic [11]. ALS was performed according to the 2015 guidelines of the American heart association (AHA). When VF was seen, rapid defibrillation was performed, and if VF persisted despite three attempts of defibrillation, 300 mg of amiodarone mixed with 5% dextrose 30 cc were administered to the patient under the emergency physician’s medical direction. If VF persisted after 300 mg of amiodarone was injected, an additional 150 mg of amiodarone was administered.

This study was approved by the Institutional Review Board (IRB) of Seoul National University Bundang Hospital (IRB approval number: B-1904-537-103) and reported according to the STROBE (Strengthening the Reporting of Observational Studies in Epidemiology) guidelines for reporting observational trials [12].

### Patients enrollment and outcomes

Patients who presented with initial ventricular fibrillation and subsequent RVF were included. RVF was defined as VF that was not terminated after three defibrillation attempts. Patients younger than 18 years and those whose cardiac arrest was caused by non-medical reasons were excluded.

The primary outcome was the neurological outcome at hospital discharge according to the time elapsed until amiodarone administration, and the secondary outcomes were the prehospital return of spontaneous circulation (ROSC), total ROSC, survival at admission, and survival to discharge according to the time elapsed until amiodarone administration.

The Good-CPC group was defined as those patients whose cerebral performance category (CPC) score was 1 or 2 at hospital discharge, and the Poor-CPC group included those patients who had a CPC of 3–5 at hospital discharge. The CPC score was assessed by emergency physicians at hospital discharge.

### Data collection

We collected Utstein variables, including age, gender, pathogenesis, arrest location, witnessed arrest, first monitored rhythm, bystander cardiopulmonary resuscitation (CPR), response time, defibrillation time, number of shocks, call-to-amiodarone administration interval, call-to-epinephrine administration interval, prehospital ROSC, survival at hospital arrival, any ROSC, survival at admission, and survival to discharge.

Pathogenesis was defined as the most likely primary cause of the cardiac arrest and was recorded as medical/traumatic cause/drug overdose/drowning/electrocution/asphyxia/not recorded. The arrest location was defined as the specific location where the event occurred or where the patient was found. It was recorded as public or non-public. The first monitored rhythm was defined as the first cardiac rhythm present when the monitor or defibrillator was attached to the patient after a cardiac arrest, and it was recorded as VF/pulseless VT/PEA/asystole. Bystander CPR was defined as CPR performed by a person who did not respond as part of an organized emergency response system to a cardiac arrest. The response time was defined as the time interval between the incoming call and the time when the first emergency response vehicle stopped at a point closest to the patient’s location. The defibrillation time was defined as the time interval between the incoming call and the time when the first shock was delivered. The number of shocks was defined as the number of shocks delivered [13].

Prehospital ROSC was defined as the achievement of ROSC at any point during the prehospital resuscitation attempt, and any ROSC was defined as the achievement of ROSC at any point during the entire resuscitation attempt. Survival at hospital arrival was defined as the patients’ being alive upon arrival at the hospital, and survival to discharge was defined as the patients’ being alive when they were discharged.

The call-to-amiodarone administration interval and the call-to-epinephrine administration interval were defined as the time intervals between the incoming call and the time each drug was first administered.

### Statistical power calculation

Statistical power analysis was performed using G*power 3.1 on a good neurological outcome at discharge rate for a two-tail logistic regression. Given the sample size of 134, a type 1 error of 0.05, good neurological outcome at discharge rates of 22.4 and 4.7% for the call-to-amiodarone administration intervals of ≤20 min and > 20 min, respectively, the statistical power of 0.99 was calculated.

### Statistical analysis

Statistical analyses were performed using SPSS software for Windows (V.20.0 K, SPSS, Chicago, IL, USA). Nominal data were presented as frequencies and percentages; continuous variables were presented as the mean and standard deviation (SD) and median and interquartile range (IQR) after assessments for normality using the Shapiro-Wilk test. The chi-square test or Fisher’s exact test was used for comparisons of nominal variables, while the independent t-test and the Mann-Whitney U test were used to compare continuous variables. *P*-values less than 0.05 were considered statistically significant.

Univariate logistic regression analysis was performed to identify the relation between the probability of a good neurological outcome at hospital discharge and the time elapsed between the incoming call and amiodarone administration.

Multivariate logistic regression analysis was performed to identify independent factors of neurological outcome at hospital discharge, as measured by the estimated odds ratio (OR) with 95% confidence intervals (CIs). Age, sex, public place, witnessed arrest, bystander CPR, targeted temperature management (TTM), the call-to-epinephrine administration interval, and the call-to-amiodarone administration interval were included in the multivariable logistic regression analysis. Variables with a *p*-value of less than 0.2 on univariate analyses, as well as clinically relevant variables were entered into the forward stepwise multiple logistic regression models.

The receiver operating characteristic (ROC) curve was used to obtain the optimal cut-off value of the call-to-amiodarone administration interval and call-to-epinephrine administration interval. According to these values, the patients were divided into two groups.

## Result

**Fig. 1:**
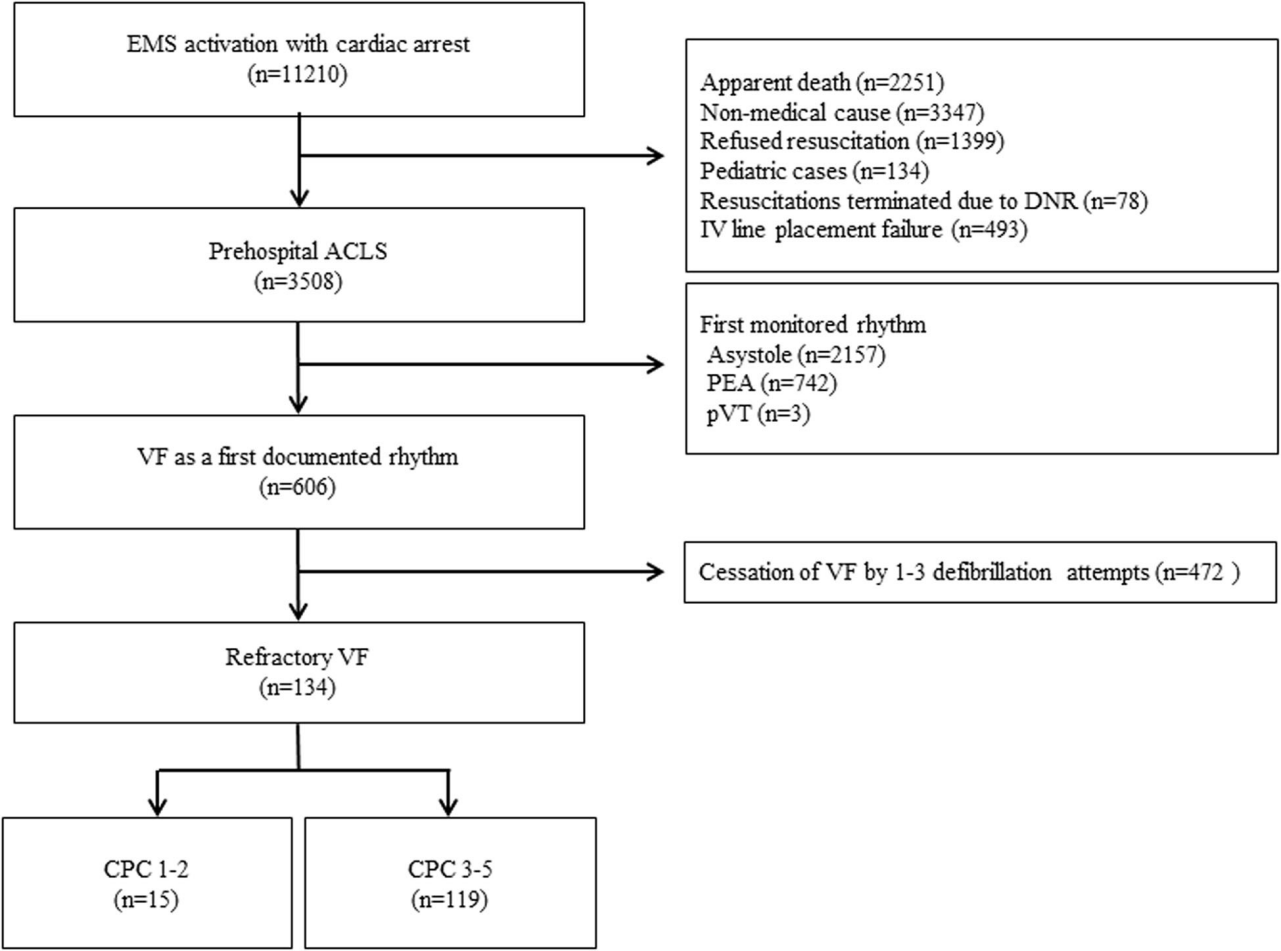
Study inclusion and exclusion. EMS: emergency medical services, ACLS: advanced cardiac life support, PEA: pulseless electrical activity, pVT: pulseless ventricular tachycardia, VF: ventricular fibrillation, CPC: cerebral performance category

There were 11,210 cardiac arrest patients during the study period, and a total of 3508 patients were eligible for prehospital drug administration. Among them, 606 showed initial VF rhythm, and 134 patients who did not respond to three or more electrical defibrillation attempts were included in this study (Fig. 1).

**Fig. 2:**
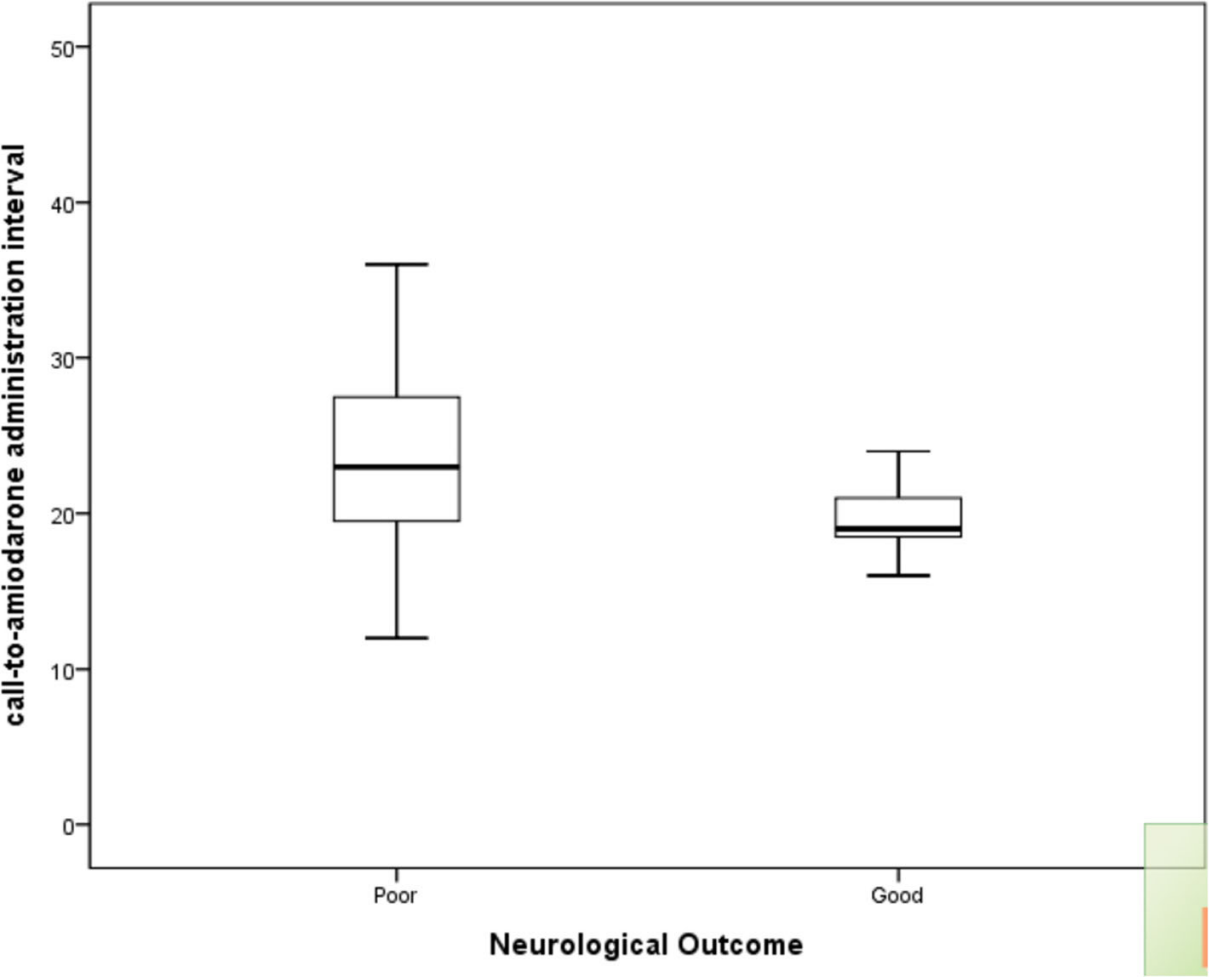
Box-Whisker plot of the call-to-amiodarone administration interval

The patients were divided into two groups according to the neurological outcome at hospital discharge. Age (51[36–65 IQR] vs 61[49–84 IQR], *p* = 0.009) and the call-to-amiodarone administration interval (19 [18–22 IQR] vs 23 [19–28 IQR], p = 0.009) showed statistical significance (Table 1; Fig. 2).

**Table 1:** Clinical and EMS^a^ characteristics

Characteristics	Total (*n* = 134)	Neurological outcome at hospital discharge		*p*-value
		Good-CPC^b^ group (*n* = 15)	Poor-CPC^b^ group (*n* = 119)	
Age, median (IQR^c^)	60 (48–71)	51 (36–65)	61 (49–84)	0.009
Male, n (%)	114 (85.1)	14 (93.3)	100 (84.0)	0.343
Hypertension, n (%)	33 (24.6)	5 (33.3)	28 (23.5)	0.524
Diabetes, n (%)	24 (17.9)	1 (6.7)	23 (19.3)	0.306
Cerebrovascular disease, n (%)	3 (2.2)	0 (0)	3 (2.2)	1.000
Heart disease, n (%)	26 (19.4)	2 (13.3)	24 (20.2)	0.735
Arrest location - Public space, n (%)	56 (41.8)	9 (60.0)	47 (39.5)	0.131
Witnessed arrest, n (%)	91 (67.9)	11 (73.3)	80 (67.2)	0.774
Bystander CPR^d^, n (%)	105 (78.4)	14 (93.3)	91 (76.5)	0.137
Response time (minutes)	7 (6–9)	7 (6–11)	7 (6–15)	0.441
Defibrillation time (minutes)	11 (9–13)	10 (9–17)	11 (9–20)	0.205
Number of shocks	7 (5–9)	6 (4–7)	7 (5–9)	0.421
TTM^e^, n(%)	15 (11.2)	6 (40.0)	9 (7.6)	0.002
Call-to-amiodarone administration interval (minutes)	23 (19–26.3)	19 (18–22)	23 (19–28)	0.009
Call-to-epinephrine administration interval (minutes)	18 (15–22)	17 (15–19)	18 (15–22)	0.113

^a^*EMS* Emergency medical service, ^b^*CPC* Cerebral performance category, ^c^*IQR* Interquartile range, ^d^*CPR* Cardiopulmonary resuscitation, ^e^*TTM* Targeted temperature management

**Fig. 3:**
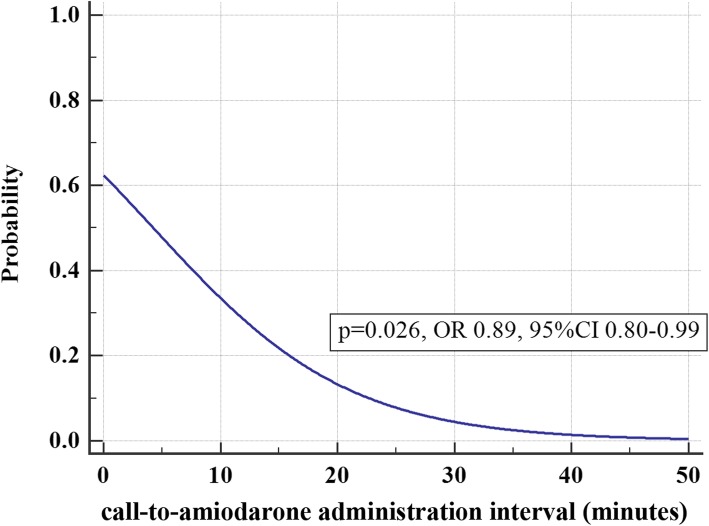
Univariate logistic regression for the probability of good neurological outcome at hospital discharge according to the call-to-amiodarone administration interval. OR: odds ratio, CI: confidence interval

In univariate logistic regression, the probability of a good neurological outcome at hospital discharge decreased as the call-to-amiodarone administration interval increased (OR 0.89 [95% CI = 0.80–0.99]) (Fig. 3). The optimal cut-off value of the call-to-amiodarone administration interval was obtained using the ROC curve analysis (AUC 0.707, 95% CI 0.622–0.782, *p* < 0.001), and the patients were divided into two groups, according to the optimal cut-off value (≤ 20, sensitivity 73.33, specificity 68.07). As a result, there were significant differences between the two groups regarding prehospital ROSC, survival at hospital arrival, any ROSC, survival at admission, survival to discharge, and good CPC at hospital discharge (Table 2).

**Table 2:** OHCA^a^ outcomes according to the call to amiodarone time

Outcomes	Total (n = 134)	Call-to-amiodarone administration interval	Call-to-amiodarone administration interval	*p*-value
		≤20 min (*n* = 49)	> 20 min (*n* = 85)	
Prehospital any ROSC^b^, n (%)	48 (35.8)	23 (46.9)	25 (29.4)	0.042
Survival at hospital arrival	26 (19.4)	15 (30.6)	11 (12.9)	0.013
Any ROSC^b^, n (%)	55 (41.0)	26 (53.1)	29 (34.1)	0.025
Survival admission, n (%)	37 (27.6)	22 (44.9)	15 (17.6)	0.001
Survival to discharge, n (%)	24 (17.9)	14 (28.6)	10 (11.8)	0.019
Good CPC^c^ at hospital discharge, n (%)	15 (11.2)	11 (22.4)	4 (4.7)	0.004

^a^*OHCA* Out-of-hospital cardiac arrest, ^b^*ROSC* Return of spontaneous circulation, ^c^*CPC* Cerebral performance category

**Fig. 4:**
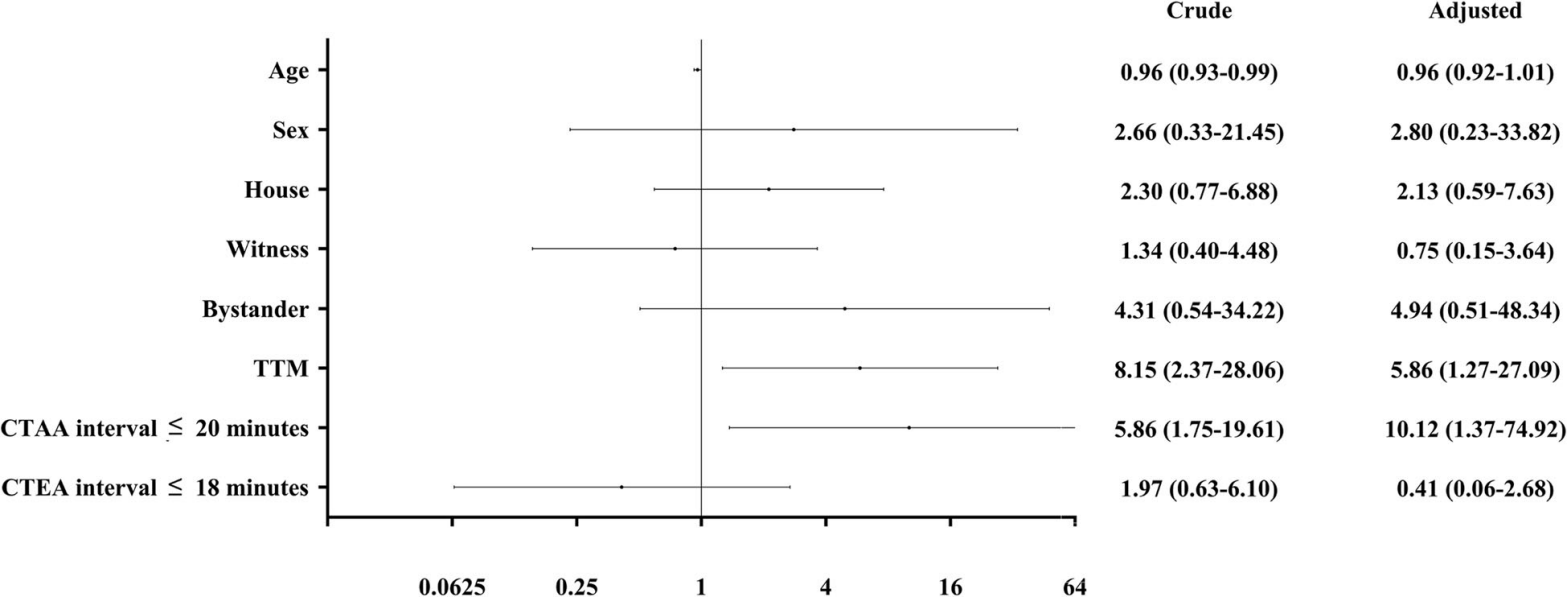
Multivariate logistic regression for the probability of good neurological outcome at hospital discharge. TTM: targeted temperature management, CTAA: call-to-amiodarone administration, CTEA: call-to-epinephrine administration

In multivariate logistic regression, TTM (OR 5.86, 95% CI 1.27–27.09) and the call-to-amiodarone administration interval ≤ 20 min (OR 10.12, 95% CI 1.37–74.92) were the independent factors affecting the neurological outcome at hospital discharge (Fig. 4).

## Discussion

Early amiodarone administration (call-to-amiodarone administration interval ≤ 20 min) was an independent factor related to good CPC at discharge in OHCA patients who showed initial VF and subsequent RVF.

There have been some studies regarding amiodarone and OHCA outcomes. Kudenchuk et al. reported that amiodarone use in OHCA patients with refractory ventricular arrhythmias resulted in a higher rate of survival to hospital admission [14]. Dorian et al. reported that, compared with lidocaine, amiodarone led to substantially higher rates of survival at hospital admission in patients with shock-resistant out-of-hospital ventricular fibrillation [15].

Similarly, a recent large randomized trial reported that epinephrine administration increases the 30-day survival rate [16]. However, according to a study on the epinephrine administration time by Hayashi et al., the neurological outcome could be improved if epinephrine were administered within 10 min [17].

Since there was no study on the effect of amiodarone administration time on OHCA outcomes, we evaluated the call-to-amiodarone administration interval to investigate its effect on neurological outcomes in RVF patients. As a result, early amiodarone administration revealed good neurological outcomes at hospital discharge.

VF increases myocardial oxygen demand [18]. Therefore, delayed defibrillation leads to progressive energy imbalance and causes profound intramyocardial acidosis, along with the depletion of high-energy nucleotides [19]. An animal experiment showed that greater hypercarbic acidosis or hypoxia required the highest dose of electrical energy for defibrillation and led to a greater number of failed resuscitation attempts [20]. Therefore, the AHA guidelines emphasize early defibrillation [21, 22].

Intramyocardial acidosis and end-organ damage may result from prolonged hypoperfusion [14] and may progress over time. For this reason, early amiodarone administration is aimed to facilitate the restoration and maintenance of a spontaneous perfusing rhythm in concert with the shock termination of VF [15], showing improvement in the neurological outcome at hospital discharge. Moreover, this result is also consistent with the 2018 AHA guidelines, which state that amiodarone may be particularly useful for those who are administered the drug early on [23].

Some studies reported that rapid defibrillation increased the rate of survival to hospital discharge [2, 24–26]. In this study, however, no difference was observed regarding the defibrillation time between the Good-CPC group and the Poor-CPC group. Given that RVF is not terminated by electrical defibrillation as a definition, this result could be acceptable.

Amiodarone was supposed to be given when VF persisted despite three defibrillation attempts. Because only patients who showed initial VF were enrolled in this study, the time when the amiodarone administration was decided would have been similar. Therefore, the call-to-amiodarone administration interval could have been determined mainly by response time and vascular access time. Given that no difference was observed in response time and the drugs are gated by the vascular access time, the latter may be an important factor. Therefore, when IV access is not available, alternative drug administration routes, such as intraosseous, should be considered.

There are some limitations to the present study. First, it was a retrospective study, and a six-month neurological outcome was not investigated. Second, we could not investigate the mechanisms by which the earlier amiodarone administration exerted its salutary effect in this study. However, despite the fact that the definition of RVF varies and the optimal interval between the incoming call and amiodarone administration has not been fully established yet, the result of this study remains compelling. Third, we could not investigate the adverse effects of amiodarone. Last, the treatment of patients after hospitalization was not standardized. Although the patients were treated in accordance with the 2015 AHA guidelines, post-resuscitation management followed the protocol of each institution.

## Conclusion

Early amiodarone administration (≤ 20 min) resulted in better neurological outcomes at hospital discharge for OHCA patients who presented with initial VF and subsequent RVF.

## Data Availability

Data sharing is not applicable to this article as no datasets were generated or analysed during the current study.

## References

[1] Jentzer JC, Clements CM, Wright RS, White RD, Jaffe AS (2016). Improving survival from cardiac arrest: a review of contemporary practice and challenges. Ann Emerg Med.

[2] Sasson Comilla, Rogers Mary A.M., Dahl Jason, Kellermann Arthur L. (2010). Predictors of Survival From Out-of-Hospital Cardiac Arrest. Circulation: Cardiovascular Quality and Outcomes.

[3] Maio VJD, De Maio VJ, Stiell IG, Wells GA, Spaite DW (2003). Optimal defibrillation response intervals for maximum out-of-hospital cardiac arrest survival rates. Ann Emerg Med.

[4] Stiell IG, Nichol G, Leroux BG, Rea TD, Ornato JP, Powell J (2011). Early versus later rhythm analysis in patients with out-of-hospital cardiac arrest. N Engl J Med.

[5] Yannopoulos D, Bartos JA, Raveendran G, Conterato M, Frascone RJ, Trembley A (2017). Coronary artery disease in patients with out-of-hospital refractory ventricular fibrillation cardiac arrest. J Am Coll Cardiol.

[6] Sakai T, Iwami T, Tasaki O, Kawamura T, Hayashi Y, Rinka H (2010). Incidence and outcomes of out-of-hospital cardiac arrest with shock-resistant ventricular fibrillation: data from a large population-based cohort. Resuscitation..

[7] Link MS, Berkow LC, Kudenchuk PJ, Halperin HR, Hess EP, Moitra VK (2015). Part 7: adult advanced cardiovascular life support: 2015 American Heart Association guidelines update for cardiopulmonary resuscitation and emergency cardiovascular care. Circulation..

[8] Singh BN, Vaughan Williams EM (1970). The effect of amiodarone, a new anti-anginal drug, on cardiac muscle. Br J Pharmacol.

[9] Kudenchuk Peter J., Brown Siobhan P., Daya Mohamud, Rea Thomas, Nichol Graham, Morrison Laurie J., Leroux Brian, Vaillancourt Christian, Wittwer Lynn, Callaway Clifton W., Christenson James, Egan Debra, Ornato Joseph P., Weisfeldt Myron L., Stiell Ian G., Idris Ahamed H., Aufderheide Tom P., Dunford James V., Colella M. Riccardo, Vilke Gary M., Brienza Ashley M., Desvigne-Nickens Patrice, Gray Pamela C., Gray Randal, Seals Norman, Straight Ron, Dorian Paul (2016). Amiodarone, Lidocaine, or Placebo in Out-of-Hospital Cardiac Arrest. New England Journal of Medicine.

[10] Tanaka H, Takyu H, Sagisaka R, Ueta H, Shirakawa T, Kinoshi T (2016). Favorable neurological outcomes by early epinephrine administration within 19 minutes after EMS call for out-of-hospital cardiac arrest patients. Am J Emerg Med.

[11] Lee DK, Park SM, Kim YJ, Lee CA, Jeong WJ, Kim GW (2018). CPR Guidance by an Emergency Physician via Video Call: A Simulation Study. Emerg Med Int.

[12] von Elm Erik, Altman Douglas G, Egger Matthias, Pocock Stuart J, Gøtzsche Peter C, Vandenbroucke Jan P (2007). The Strengthening the Reporting of Observational Studies in Epidemiology (STROBE) Statement: Guidelines for Reporting Observational Studies. PLoS Medicine.

[13] Perkins GD, Jacobs IG, Nadkarni VM, Berg RA, Bhanji F, Biarent D (2015). Cardiac Arrest and Cardiopulmonary Resuscitation Outcome Reports: Update of the Utstein Resuscitation Registry Templates for Out-of-Hospital Cardiac Arrest: A Statement for Healthcare Professionals From a Task Force of the International Liaison Committee on Resuscitation (American Heart Association, European Resuscitation Council, Australian and New Zealand Council on Resuscitation, Heart and Stroke Foundation of Canada, InterAmerican Heart Foundation, Resuscitation Council of Southern Africa, Resuscitation Council of Asia); and the American Heart Association Emergency Cardiovascular Care Committee and the Council on Cardiopulmonary, Critical Care, Perioperative and Resuscitation. Resuscitation.

[14] Kudenchuk PJ, Cobb LA, Copass MK, Cummins RO, Doherty AM, Fahrenbruch CE (1999). Amiodarone for resuscitation after out-of-hospital cardiac arrest due to ventricular fibrillation. N Engl J Med.

[15] Dorian P, Cass D, Schwartz B, Cooper R, Gelaznikas R, Barr A (2002). Amiodarone as compared with lidocaine for shock-resistant ventricular fibrillation. N Engl J Med.

[16] Perkins GD, Ji C, Deakin CD, Quinn T, Nolan JP, Scomparin C (2018). A randomized trial of epinephrine in out-of-hospital cardiac arrest. N Engl J Med.

[17] Hayashi Y, Iwami T, Kitamura T, Nishiuchi T, Kajino K, Sakai T (2012). Impact of early intravenous epinephrine administration on outcomes following out-of-hospital cardiac arrest. Circ J.

[18] Yaku H, Goto Y, Ohgoshi Y, Kawaguchi O, Oga K, Oka T (1993). Determinants of myocardial oxygen consumption in fibrillating dog hearts. Comparison between normothermia and hypothermia. J Thorac Cardiovasc Surg.

[19] Johnson BA, Weil MH, Tang W, Noc M, McKee D, McCandless D (1995). Mechanisms of myocardial hypercarbic acidosis during cardiac arrest. J Appl Physiol.

[20] Idris AH, Wenzel V, Becker LB, Banner MJ, Orban DJ (1995). Does hypoxia or hypercarbia independently affect resuscitation from cardiac arrest?. Chest..

[21] Berg RA, Hemphill R, Abella BS, Aufderheide TP, Cave DM, Hazinski MF (2010). Part 5: Adult Basic Life Support: 2010 American Heart Association guidelines for cardiopulmonary resuscitation and emergency cardiovascular care. Circulation..

[22] Hansen CM, Kragholm K, Granger CB, Pearson DA, Tyson C, Monk L (2015). The role of bystanders, first responders, and emergency medical service providers in timely defibrillation and related outcomes after out-of-hospital cardiac arrest: Results from a statewide registry. Resuscitation.

[23] Panchal AR, Berg KM, Kudenchuk PJ, Del Rios M, Hirsch KG, Link MS (2018). 2018 American Heart Association focused update on advanced cardiovascular life support use of antiarrhythmic drugs during and immediately after cardiac arrest: an update to the American Heart Association guidelines for cardiopulmonary resuscitation and emergency cardiovascular care. Circulation..

[24] Rea TD, Cook AJ, Stiell IG, Powell J, Bigham B, Callaway CW (2010). Predicting Survival After Out-of-Hospital Cardiac Arrest: Role of the Utstein Data Elements. Ann Emerg Med.

[25] Chan PS, Nichol G, Krumholz HM, Spertus JA, Nallamothu BK, American Heart Association National Registry of Cardiopulmonary Resuscitation (NRCPR) Investigators (2009). Hospital variation in time to defibrillation after in-hospital cardiac arrest. Arch Intern Med.

[26] Valenzuela TD, Roe DJ, Nichol G, Clark LL, Spaite DW, Hardman RG (2000). Outcomes of rapid defibrillation by security officers after cardiac arrest in casinos. N Engl J Med.

