# Correction to: Impact of early intravenous amiodarone administration on neurological outcome in refractory ventricular fibrillation: retrospective analysis of prospectively collected prehospital data

**DOI:** 10.1186/s13049-020-0718-z

**Published:** 2020-03-18

**Authors:** Dong Keon Lee, Yu Jin Kim, Giwoon Kim, Choung Ah. Lee, Hyung Jun Moon, Jaehoon Oh, Hae Chul Yang, Han Joo Choi, Young Taeck Oh, Seung Min Park

**Affiliations:** 1 Department of Emergency Medicine, Seoul National University Bundang Hospital, 1362082, Gumi-ro 173 Beon-gil, Bundang-gu, Seongnam-si, Gyeonggi-do Republic of Korea; 2 Department of Emergency Medicine, Soonchunhyang University Bucheon Hospital, 170 , Jomaru-ro, Wonmi-gu, Bucheon-si, 14584 Gyeonggi-do Republic of Korea; 3 Department of Emergency Medicine, Hallym University Dongtan Sacred Heart Hospital, 7, Keunjaebong-gil, Hwaseong-si, 18450 Gyeonggi-do Republic of Korea; 4 Department of Emergency Medicine, Soonchunhyang University Cheonan Hospital, 31, Suncheonhyang 6-gil, Dongnam-gu, Cheonan-si, 31151 Chungcheongnam-do Republic of Korea; 5 Department of Emergency Medicine, College of Medicine, Hanyang University, 222-1, Wangsimni-ro, Seongdong-gu, Seoul, 04763 Republic of Korea; 6 Researcher, Seoul National University Bundang Hospital, 82, Gumi-ro 173beon-gil, Bundang-gu, Seongnam-si, 13620 Gyeonggi-do Republic of Korea; 7 Department of emergency medicine, Dankook University College of Medicine, 201 Manghyang-ro, Dongnam-gu, Cheonan-si, 31116 Chungcheongnam-do Republic of Korea

**Correction to: Scand J Trauma Resusc Emerg Med**

**https://doi.org/10.1186/s13049-019-0688-1**

Following the publication of the original article [1], the authors unfortunately became aware of some typesetting and resolution problems in Figs. 1 and 2.

Hence, new higher resolution figures are provided here:

**Fig. 1:**
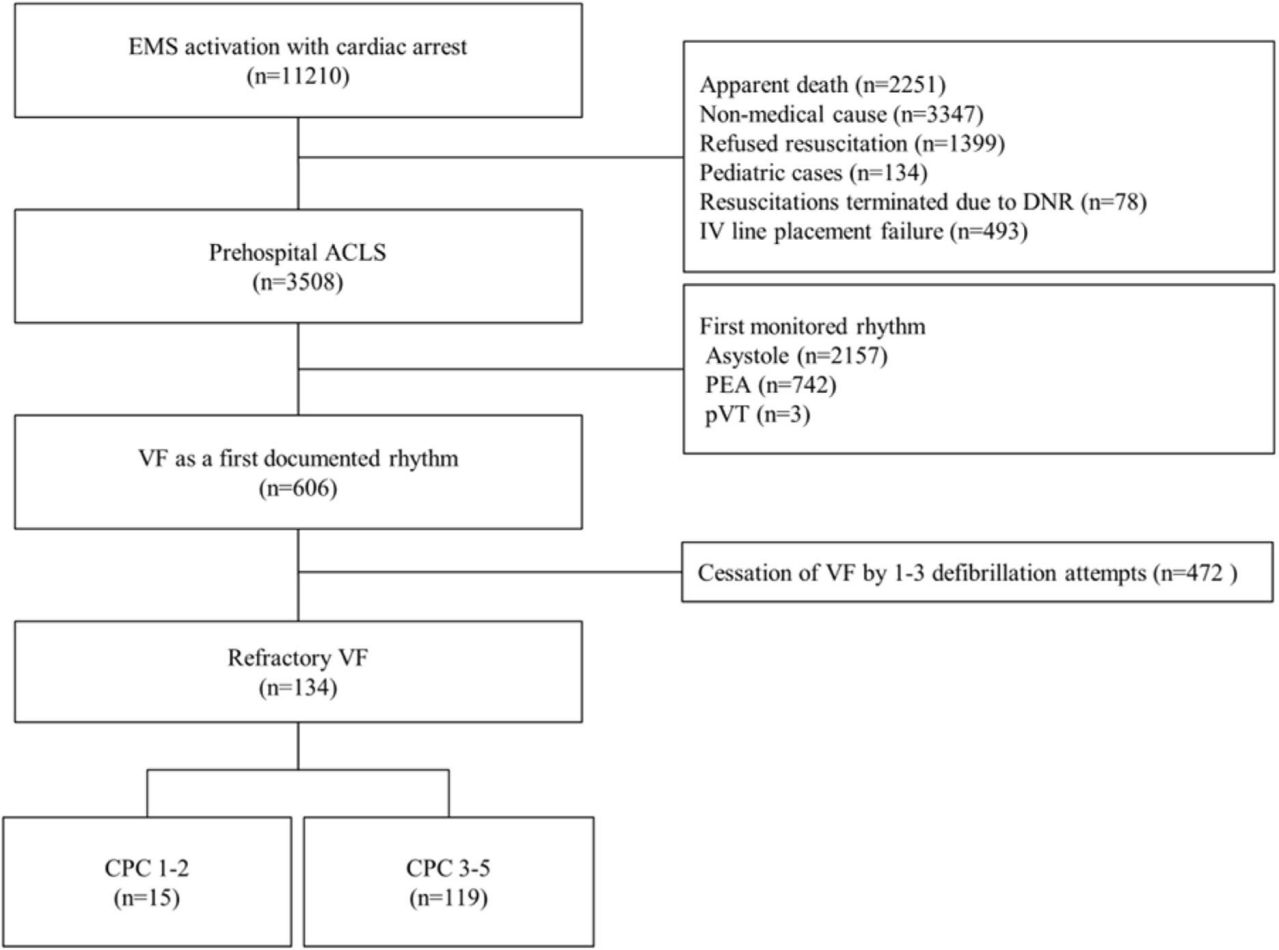
Study inclusion and exclusion. EMS: emergency medical services, ACLS: advanced cardiac life support, PEA: pulseless electrical activity, pVT: pulseless ventricular tachycardia, VF: ventricular fibrillation, CPC: cerebral performance category

**Fig. 2:**
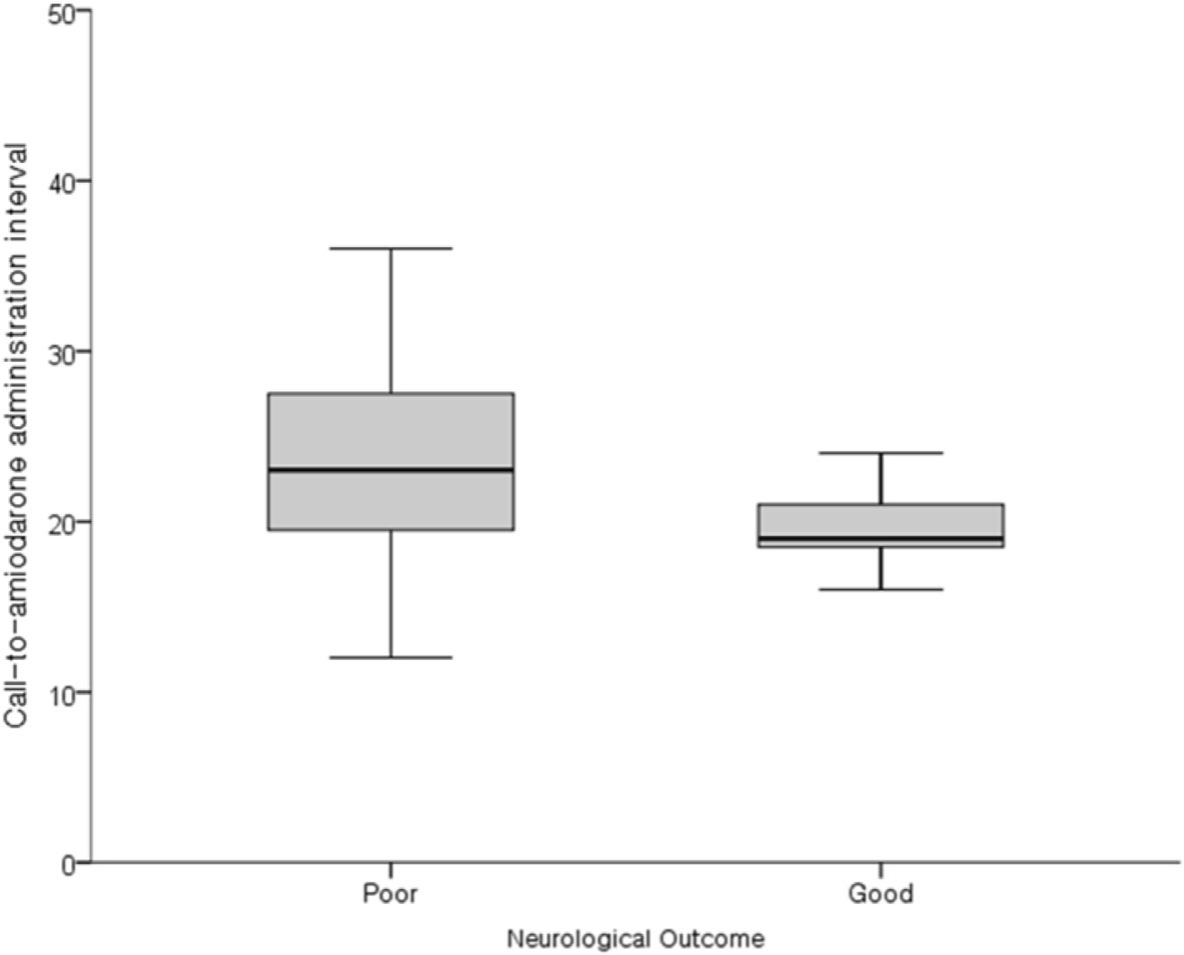
Box-Whisker plot of the call-to-amiodarone administration interval
